# Transgenerational Social Stress, Immune Factors, Hormones, and Social Behavior

**DOI:** 10.3389/fevo.2015.00149

**Published:** 2016-01-12

**Authors:** Christopher A. Murgatroyd, Jessica A. Babb, Steven Bradburn, Lindsay M. Carini, Gillian L. Beamer, Benjamin C. Nephew

**Affiliations:** 1 Centre for Healthcare Science Research, Manchester Metropolitan University, Manchester, UK; 2 Department of Anesthesia, Boston Children’s Hospital, Boston, MA, USA; 3 Department of Biomedical Sciences, Tufts University Cummings School of Veterinary Medicine, North Grafton, MA, USA; 4 Department of Infectious Disease and Global Health, Tufts University Cummings School of Veterinary Medicine, North Grafton, MA, USA

**Keywords:** social stress, social behavior, immune system, transgenerational effects, Inflammation, BDNF, progesterone, cytokines

## Abstract

A social signal transduction theory of depression has been proposed that states that exposure to social adversity alters the immune response and these changes mediate symptoms of depression such as anhedonia and impairments in social behavior The exposure of maternal rats to the chronic social stress (CSS) of a male intruder depresses maternal care and impairs social behavior in the F1 and F2 offspring of these dams. The objective of the present study was to characterize basal peripheral levels of several immune factors and related hormone levels in the adult F2 offspring of CSS exposed dams and assess whether changes in these factors are associated with previously reported deficits in allogrooming behavior. CSS decreased acid glycoprotein (α1AGP) and intercellular adhesion molecule-1 (ICAM-1) in F2 females, and increased granulocyte macrophage-colony stimulating factor (GM-CSF) in F2 males. There were also sex dependent changes in IL-18, tissue inhibitors of metalloproteinases 1 (TIMP-1), and vascular endothelial growth factor (VEGF). Progesterone was decreased and alpha melanocyte stimulating hormone (α-MSH) was increased in F2 males, and brain-derived neurotrophic factor (BDNF) was decreased in F2 females. Changes in α1AGP, GM-CSF, progesterone, and α-MSH were correlated with decreased allogrooming in the F2 offspring of stressed dams. These results support the hypothesis that transgenerational social stress affects both the immune system and social behavior, and also support previous studies on the adverse effects of early life stress on immune functioning and stress associated immunological disorders, including the increasing prevalence of asthma. The immune system may represent an important transgenerational etiological factor in disorders which involve social and/or early life stress associated changes in social behavior, such as depression, anxiety, and autism, as well as comorbid immune disorders. Future studies involving immune and/or endocrine assessments and manipulations will address specific questions of function and causation, and may identify novel preventative measures and treatments for the growing number of immune mediated disorders.

## INTRODUCTION

A social signal transduction theory of depression has been proposed that states that exposure to social adversity, especially during early life, alters immune responses, and these changes mediate depression symptoms such as anhedonia and impaired social behavior (Slavich and Irwin, 2014). Cytokine mediated effects have been specifically implicated in depression and anxiety associated alterations in social behavior (Dantzer et al., 2008). Stressors during the perinatal period can induce persistent changes in the immune system and offspring behavior (Bilbo and Schwarz, 2012). For example, maternal immune activation induces adverse changes in offspring social behavior (Hodes et al., 2014; Machado et al., 2015). Exposure to early life stress also alters the systemic immune response and behavior (O’Mahony et al., 2009, 2011) and there is recent evidence that inflammatory factors (IL-1 and IL-6) increase vulnerability to stress related disorders and are associated with impairments in social behavior (Hodes et al., 2014; Wood et al., 2014). Social defeat, a robust social stressor often used in rodent studies, increases cytokine secretion (Powell et al., 2009), deficient maternal care alters immune associated gene expression (Cole et al., 2012), and it is postulated that inflammation may mediate the adverse effects of early life stress on mental health (Danese et al., 2007; Carpenter et al., 2010).

**FIGURE 1 |:**
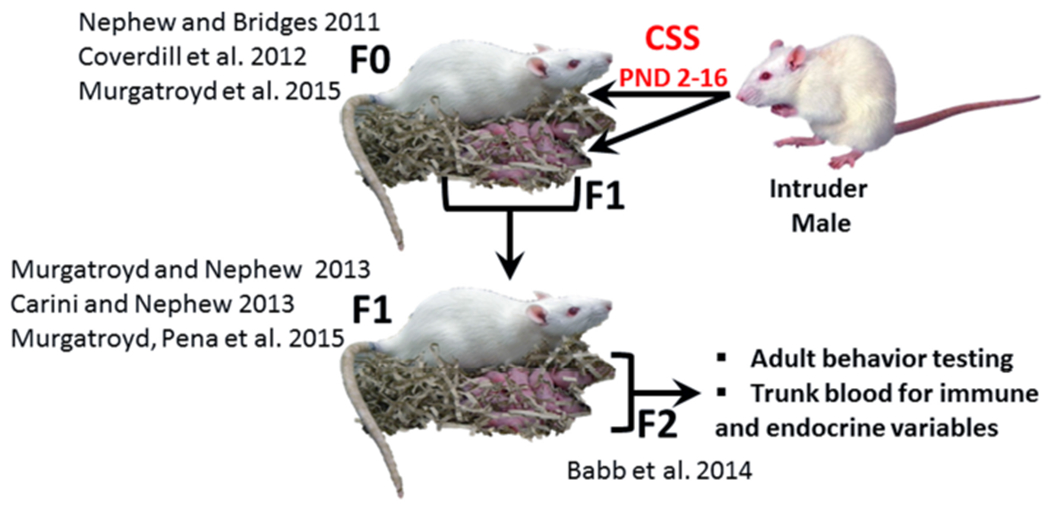
The CSS model of postpartum depression and anxiety. F0 dams are exposed to novel male intruder stress for 1 h/day during lactation days 2–15. This social stress is early life stressor for the F1 generation. Both the F0 and F1 dams exhibit depressed maternal care and increased maternal anxiety, and the depressed maternal care of the F1 dams is an early life stressor for the F2 generation. The current study focused on male and female F2 adults. (F0: Nephew and Bridges, 2011; Coverdill et al., 2012; Murgatroyd et al., 2015b; F1: Carini and Nephew, 2013; Murgatroyd and Nephew, 2013; Murgatroyd et al., 2015a; F2: Babb et al 2014).

Exposure to early life stress, both prenatal and neonatal, can induce robust, behaviorally relevant changes in brain development (Howerton and Bale, 2012; Bale, 2015), and it has been suggested that these effects may be immune mediated (Howerton and Bale, 2012). Neonatal endotoxin treatment affects the development of the stress response and may increase susceptibility to stress related disorders (Shanks et al., 1995) and several additional studies have supported the hypothesis that the adverse effects of early life adversity on neural plasticity are mediated by inflammation (Musaelyan et al., 2014). The chronic social stress (CSS) model of postpartum depression and anxiety (Figure 1) induces substantial changes in the maternal behavior of F0 rat dams exposed to chronic male intruder stress (interaction with a novel male intruder during days 2–16 of lactation; Nephew and Bridges, 2011; Carini and Nephew, 2013; Carini et al., 2013; Murgatroyd et al., 2015b) and also disrupts maternal care in F1 dams (Carini and Nephew, 2013; Murgatroyd and Nephew, 2013; Murgatroyd et al., 2015a) and social behavior in juvenile and adult F2 offspring (Babb et al., 2014). For the F1 and F2 offspring of stressed dams, the effects of CSS on social behavior may be mediated by early life exposure to depressed F0 maternal care and/or the male intruder stressor (F1 offspring) or depressed F1 maternal care (F2 offspring). The social behavior of both male and female F2 offspring of CSS exposed dams is disrupted, with a decrease in allogrooming during adult social interactions (Babb et al., 2014), supporting the use of the CSS model to study the transgenerational effects of stress on the etiology of disorders that involve maladaptive changes to social behavior, such as depression, anxiety, and autism.

The objective of the present study was to characterize basal peripheral levels of immune factors and related hormone levels in the adult F2 offspring of CSS exposed dams and assess whether changes in these factors are associated with previously reported alterations in social behavior. The F2 generation was chosen for this study due to the observation of significant social deficits (most notably allogrooming) in these animals (Babb et al., 2014), similar to recent work on IL-6 and social behavior in rodent studies of resilience to social stress (Hodes et al., 2014). Due to the novelty of this research topic in the context of the CSS model, we targeted a broad panel of immune factors as well as progesterone, brain derived neurotrophic factor (BDNF), and alpha melanocyte stimulating hormone (α-MSH) which comprise interacting networks that are not fully understood (Lipton and Catania, 1997; Luger and Brzoska, 2007; Tait et al., 2008; Calabrese et al., 2014). The specific immune factors and hormones were chosen based on a review of applicable rodent and clinical literature, previous published data from the CSS model, and discussions with several basic and clinical immunologists. Given the evidence implicating exposure to social stress and immune factors in altered social behavior, it was hypothesized that basal immune factors, progesterone, BDNF, and α-MSH levels would be altered in CSS F2 offspring and that these changes would be associated with decreased social behavior.

## METHODS

### Animals

Sprague-Dawley rats in this study were maintained in accordance with the guidelines of the Committee of the Care and Use of Laboratory Animals Resources, National Research Council, and the research protocol was approved by the Tufts Institutional Animal Care and Use Committee. Food and water were provided *ad libitum*, and light cycle was 12L/12D, with lights on at 0700. All F0 dams were purchased as adults (Charles River, Wilmington, MA), mated with breeder males, and housed in groups of three until the day prior to parturition. Litters were culled to five males and five females on the day of parturition. The F0 sample sizes were 11 control dams and 13 CSS dams.

### CSS Model

See Figure 1 for a visual overview of the CSS model. The F0 CSS dams were subjected to a CSS protocol from postnatal days (PND) 2 to 16 as reported previously (Nephew and Bridges, 2011; Carini et al., 2013). This procedure consisted of placing a similarly sized (220–300 g) novel Sprague Dawley male intruder into a lactating female’s home cage (10.5″W × 19″D × 8″H) for 1 h from PND 2 to 16. The F1 pups were left in the cage during the intruder presentation. Control dams were not exposed to the CSS protocol; they were only tested for maternal care and maternal aggression on PND days 2, 9, and 16 to allow for behavioral comparisons with the CSS exposed animals. Behavioral, endocrine, and neural gene expression data from these F0 dams has been published (Murgatroyd et al., 2015b).

### F1 and F2 Offspring

F1 CSS females were the offspring of F0 dams exposed to CSS. F1 control females were the offspring of the F0 control dams who were not exposed to CSS. Thus, the differences in the F1 generations were presence (F1 CSS) or absence (F1 control) of attenuated maternal care and conflict between F0 dams and the male intruders during age 2–16 days. After day 16, the F1 CSS and F1 controls were treated identically. After weaning all F1 pups on day 23, the female offspring from the 12 control and 12 CSS dams were housed in groups of four until 70 days of age when two from each litter were mated with six proven breeder males from Charles River (24 F1 females for the control and CSS groups). Behavioral and endocrine data from those F1 females have been previously reported (Carini and Nephew, 2013; Murgatroyd and Nephew, 2013; Murgatroyd et al., 2015a).

Total F2 pup number and litter weights were recorded on the day of parturition, and litters were then culled to five females and five males. The control F2 and CSS F2 animals were treated identically throughout the study; the only difference between the two groups was the attenuated maternal care (including deficits in pup grooming, nursing, and milk intake by pups) and increased restlessness and anxiety-related behavior (nesting, self-grooming, locomotor activity) expressed by the CSS F1 dams toward the F2 offspring. To summarize, the early life stress experience of the F2 generation consisted of exposure to attenuated maternal care from the F1 dams. The final F2 adult sample sizes were 10 for the control groups and 13 for the CSS groups, and there were no treatment differences in litter size or number or bodyweights at the juvenile, or adult stage, (all *p* > 0.2). Juveniles were euthanized at 42 days old, and adults were euthanized at 72 days.

### Adult F2 Social Behavior Testing

The experimental rat was removed from the home cage and placed in a clean breeding cage (16 × 20 × 8 min.) for 10 min to allow for locomotor acclimation to the novel environment. An empty clear plastic mouse cage covered with a plastic mesh top was then placed in the breeding cage for 10 min, and a randomly selected same sex novel rat from the same treatment group was placed under the cage top to test for social approach for 10 min. At the end of the 10 min of social approach recording, the mouse cage top was removed, and the focal and novel animals were allowed to interact for 10 min. Behaviors scored for social approach consisted of time spent near and distant from the novel rat (area next to the mouse top was divided into two sections), time spent on top of the mouse cage top, olfactory investigation of the novel rat through the mesh of the mouse cage cover, self grooming, and total social approach (the sum of time near novel rat, on top of mouse cage top, and olfactory investigation). Adult social behaviors scored consisted of rostral and caudal investigation, lateral contact, dorsal contact, tail grabbing, allogrooming, self grooming, locomotor activity, aggression, and total social contact (the sum of investigation, contact, tail grabbing, and allogrooming). The social behavior data from the animals in this study have been previously reported in *Hormones and Behavior* (Babb et al., 2014), and only the novel cytokine data and cytokine/allogrooming correlations will be presented in the current study. During the social interaction test, adult F2 males and females spent more time investigating a novel conspecific and less time in direct social interaction, as assessed by time spent allogrooming (Babb et al., 2014). The current decrease in F2 adult allogrooming is supported by decreased pup grooming in F2 dams which were littermates of the animals in the present study (unpublished data). All experimental animals were euthanized within 3 min of entering the animal room between 0800 and 1000 the day following social behavior testing, and trunk blood was collected for the analysis of basal immune and hormone levels.

### Immune and Endocrine Assays

An assay panel of pro- and anti-inflammatory cytokines (CXCL3, CXCL2, GM-CSF, sICAM-1, IFNγ, IL-1α, IL-1β, IL-2, IL-4, IL-6, IL-10, IL-13, IL-18, L-Selectin, TIMP-1, TNFα, VEGF) from R&D Systems (R&D Systems, UK) was conducted on a Luminex 200 Bio-Plex Platform. Immediately prior to the initiation of the study, the Bio-Plex platform underwent a complete on-site maintenance cycle. Samples were thawed directly on the day of analysis. Working wash solutions and protein standards were prepared within 1 h of beginning the assay by reconstituting the standard in assay diluent and performing serial dilutions according to manufacturer specifications. A magnetic plate washer was utilized during the plate washing stages. Following processing, protein concentrations were calculated and analyzed with the xPONENT software (Luminex, v.3.1.871). Additional cytokines and endocrine targets, IgE, CRP, α1AGP, BDNF, progesterone, and α-MSH were measured by individual ELISAs (R&D Systems, U.S.). Samples were run in duplicate in an individual assay to eliminate interassay variation, and intraassay variability was of 3–7%.

### Statistics

Basal cytokine and hormone levels were analyzed with two-way ANOVA (treatment and sex), which were followed with individual two-tailed *t*-tests within each sex (*t*-tests were not corrected for multiple comparisons). Pearson correlations were used to test for significant cytokine and hormone behavioral associations on the combined male and female data from the control and CSS F2 groups, as well as separate tests on combined control and CSS data from each sex in targets where there was an effect of CSS.

## RESULTS

**FIGURE 2 |:**
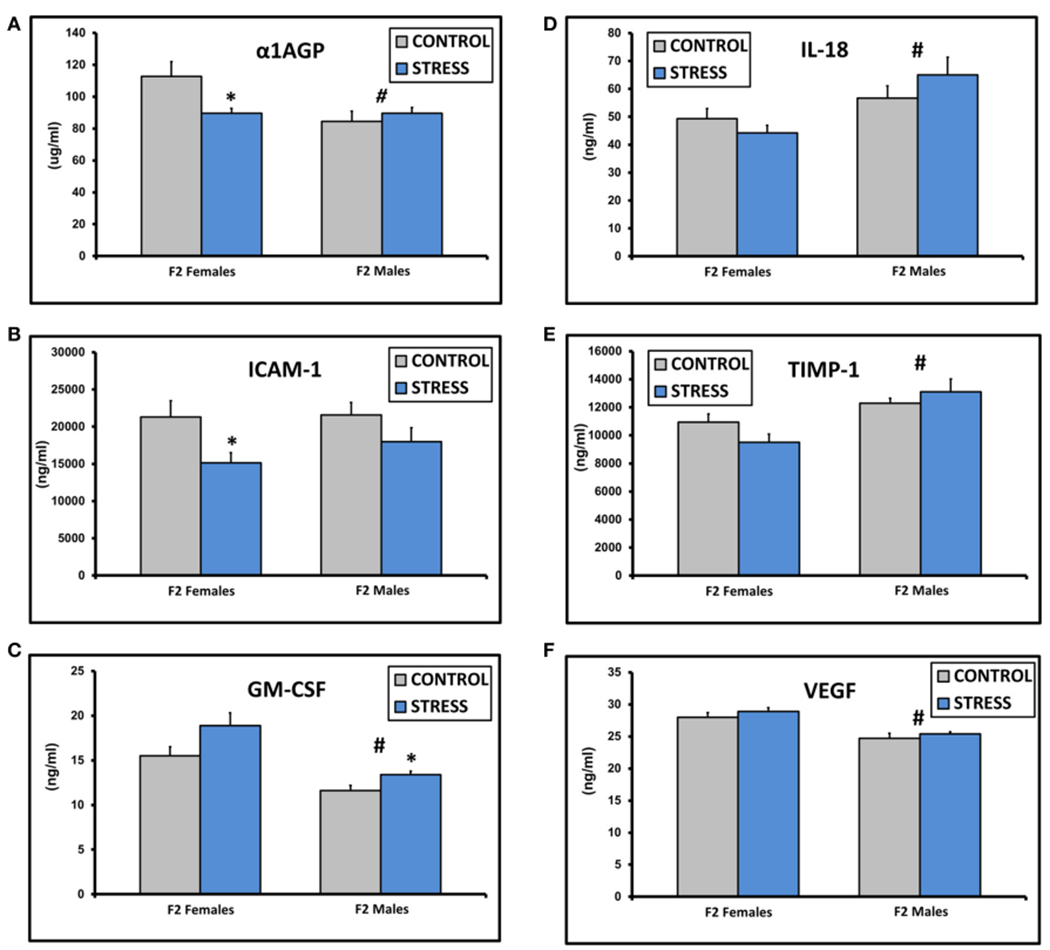
Mean ± SEM basal plasma levels of α-1AGP (A), ICAM-1 (B), GM-CSF (C), IL-18 (D), TIMP-1 (E), and VEGF (F) in the adult F2 male and female offspring of control dams and dams exposed to chronic social stress during lactation. *Denotes significant effect of CSS treatment, #denotes significant effect of sex (*p* < 0.05).

The assay values for CXCL2, IFNγ, IL-1β, IL-2, IL-4, IL-10, IL-13, and TNFα fell below the standard curve of the Luminex ELISA. There were no significant differences in the values for IgE, CRP, CXCL3, IL-1α, or L-Selectin (all *p* > 0.1). There was a significant effect of sex on α1AGP levels [*F*_(1, 45)_ = 5.0, *p* = 0.03] and a significant interaction between sex and treatment [*F*_(1, 45)_ = 6.1, *p* = 0.03, Figure 2A]. α1AGP levels were lower in CSS F2 females compared to control F2 females (*p* = 0.03, Figure 2A). There was an overall effect of treatment on soluble ICAM-1, with lower levels in CSS F2 animals [*F*_(1, 45)_ = 5.0, *p* = 0.03, Figure 2B]. ICAM-1 levels were decreased in CSS F2 females compared to controls (*p* = 0.02, Figure 2B). There were significant effects of sex [*F*_(1, 45)_ = 24.8, *p* < 0.01] and treatment (*F*_(1, 45)_ = 7.1, *p* = 0.01] on GM-CSF, with levels being generally lower in males but higher in CSS F2 males compared to control F2 males (*p* = 0. 02, Figure 2C). IL-18 [*F*_(1, 45)_ = 10.3, *p* < 0.01, Figure 2D] and TIMP-1 [*F*_(1, 45)_ = 14.9, *p* < 0.01, Figure 2E] levels were higher in F2 males compared to F2 females, where levels of VEGF were lower in F2 males [*F*_(1, 45)_ = 32.0, *p* < 0.01, Figure 2F]. There was also a non-significant trend for increased levels of IL-6 in the CSS adult F2 females [2023.2 ± 324.2 vs. 1223.1 ± 246.0, *F*_(1, 22)_ = 3.9, *p* = 0.06].

**FIGURE 3 |:**
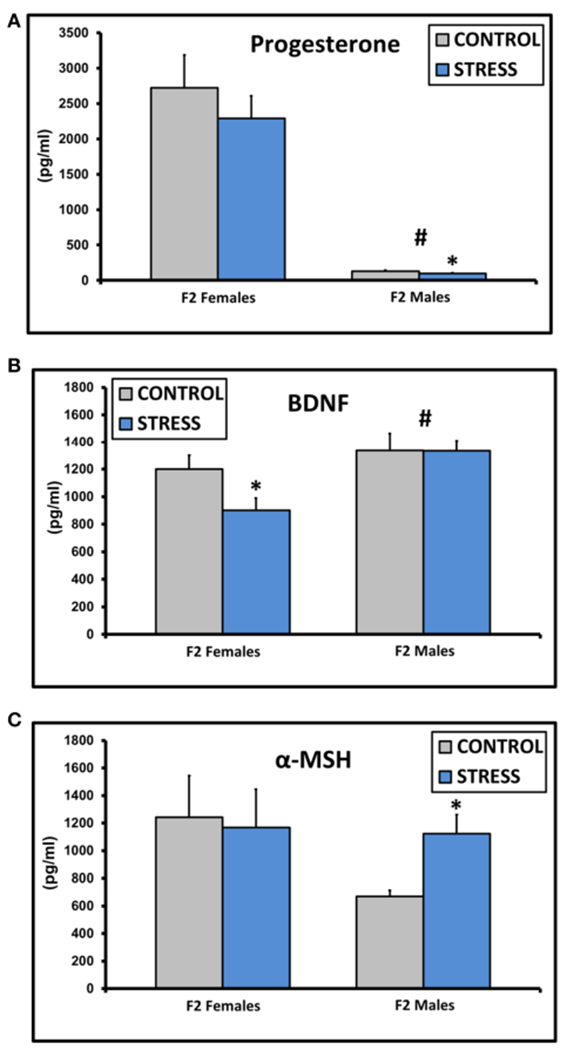
Mean ± SEM basal plasma levels of progesterone (A), BDNF (B), and α-MSH (C) in the adult F2 male and female offspring of control dams and dams exposed to chronic social stress during lactation. *Denotes significant effect of CSS treatment, #denotes significant effect of sex (*p* < 0.05).

Progesterone levels were decreased in males [*F*_(1, 45)_ = 75.5, *p* < 0.01], and levels in CSS F2 males (97.0 ± 10.8) were lower compared to control F2 males (129.4 ± 12.1, *p* < 0.05, Figure 3A). BDNF levels were higher in males [*F*_(1, 45)_ = 8.6, *p* < 0.01], and CSS F2 females had lower BDNF levels than control F2 females (*p* = 0.03, Figure 3B). α-MSH levels were higher in CSS F2 males compared to control F2 males (*p* < 0.01, Figure 3C).

α1AGP levels were positively correlated with allogrooming in all groups combined as well as specifically in female control and CSS F2 animals (Table 1). GM-CSF and α-MSH were negatively associated with allogrooming in males, and progesterone was positively associated with allogrooming in all groups combined (Table 1).

## DISCUSSION

Recent interest in the role of the immune system in behavior and the pathophysiology of stress associated psychiatric disorders stimulated the present investigation of the effects of CSS on peripheral immune factors and related hormones in the F2 offspring of CSS exposed dams. There is growing interest in the interactions between the immune system and neurohormonal signaling, and relevant behavioral models are needed to determine the mechanisms of these interactions in the context of disease pathology. The CSS paradigm had transgenerational sex specific effects in the F2 generation on basal α1AGP, ICAM-1, GM-CSF, progesterone, BDNF, and α-MSH. In contrast, while there were overall sex differences in IL-18, TIMP-1, and VEGF, these factors were not affected by CSS. Changes in α1AGP, GM-CSF, progesterone, and α-MSH were associated with allogrooming in a social interaction test. While it is possible that the social behavior testing had an effect on the immune factor and hormone levels collected the next day, the data can be considered basal in terms of the lack of any acute behavioral or immune (LPS, infection, etc.) challenge prior to collection. The basal state of the samples is supported by previously published F2 adult corticosterone and prolactin levels from the animals in the current study (Atkinson and Waddell, 1997; Babb et al., 2014). The data support the hypothesis that social stress has extensive sex specific transgenerational effects on the immune and endocrine systems which are associated with changes in social behavior. Given the design of the CSS paradigm, it is unknown if these effects are due to early life stress exposure of the F1 dams, early life stress exposure of the F2 animals, or a combination. It is postulated that the changes in immune factors, hormones, and social behavior may be the result of additive epigenetic influences in successive generations (Lyall et al., 2014).

Alpha 1 acid glycoprotein (α1AGP), a major acute phase reactant secreted into the plasma by the liver, is believed to act as an anti-inflammatory and immunomodulatory agent with increased plasma levels occurring during tissue injury, inflammation and infection (Fournier et al., 2000). In male rats, acute stress such as tail shock elevates α1AGP (Deak et al., 1997). In humans, α1AGP levels are associated with exposure to psychological stress (Maes et al., 1997) and depression (Nieto et al., 2000). The current data show decreased α1AGP in CSS F2 females, and this attenuation is associated with a related decrease in allogrooming that is driven by the effects of CSS in females. These data indicate that general disruptions in the inflammatory pathway due to early life stress, in either direction, may be associated with behavioral effects. In support of this hypothesis, the direction of changes in immune factors has been linked to coping strategy in rodent models, where passive coping is associated with pro-inflammatory processes, and active coping and resistance to stress related pathology is associated with inflammatory suppression (Finnell et al., 2015; Wood et al., 2015). Given the available behavioral data from the CSS F2 adult animals, it is difficult to speculate on their stress coping strategy or resistance to stress related pathology. It is also possible that there are sex specific α1AGP responses to stress in rats, which is supported by the present data.

A member of the immunoglobulin superfamily of adhesion receptors, intercellular adhesion molecule-1 (ICAM-1) is involved the generation of the immune response and is a primary marker of immune activation (Boyd et al., 1988), and plasma concentration of ICAM-1 are a biomarker of infection and prognosis in patients with various chronic inflammation-related diseases (de Pablo et al., 2013). Similar to α1AGP, ICAM-1 levels were decreased in CSS F2 females. This decrease in both pro and anti-inflammatory factors suggests that CSS has a general suppressive effect on the production of immune factors. While there are not many studies on the effects of early life stress specifically on ICAM-1, basal cell carcinoma patients that were maltreated by their parents exhibited poor ICAM-1 responses (Fagundes et al., 2012). A lack of significant association with allogrooming indicates that it may not mediate allogrooming in the CSS paradigm. Alternative roles for the CSS induced change in ICAM-1 include the regulation of inflammatory responses to disease related immune challenges, and ongoing CSS studies are investigating the effects of CSS on responses to immune challenges.

Granulocyte macrophage-colony stimulating factor (GM-CSF) is a hematopoietic growth factor with pro-inflammatory functions and mediates the adverse effects of social stress on inflammatory pathways (Powell et al., 2013). This cytokine may mediate global changes in inflammatory responses in stressed animals, as exposure to repeated social stress prior to allergen inhalation worsens airway inflammation in rodents, including levels of GM-CSF (Bailey et al., 2009). In addition, resident intruder stress and anhedonia have recently been associated with elevated levels of GM-CSF in the locus coeruleus (Finnell et al., 2015), and the current elevated basal levels in CSS F2 males could represent a transgenerational behavioral effect of a similar social stress through GM-CSF given that the F2 animals were not directly exposed to CSS. Children with autism spectrum disorder (ASD) exhibit a trend for elevated GM-CSF compared to healthy controls (Ashwood et al., 2011), social deficits are reported in ASD and in the CSS F2 animals (Babb et al., 2014), and early life stress in mothers is associated with an increased risk for autism in offspring (Roberts et al., 2013, 2014). Both animal and human studies of GM-CSF indicate that it may be involved in the adverse behavioral effects of social stress, but it is unknown if changes in this cytokine mediate changes in F2 social behavior or are an inflammatory indicator of exposure to social stress.

The matrix metalloproteinases and their inhibitors (tissue inhibitors of metalloproteinases, TIMP) mediate the remodeling of the pericellular environment and have been postulated to regulate methamphetamine induced sensitization and reward through modulation of extracellular dopamine (Mizoguchi et al., 2008). While we did not see effects of CSS on basal levels of TIMP-1, increased levels in males may reflect sex-dependent responses to stress and/or reward. There was also a sex difference in basal VEGF, and while most of the focus on stress, depression, and neuroplasticity has been directed toward BDNF, VEGF is also a potent mediator of neuroplasticity (Duman and Monteggia, 2006; Pittenger and Duman, 2007). It is possible that there were effects of the CSS on basal VEGF in the F2 animals at earlier life history stages (infant, juvenile) that mediated alterations in neural development only during those periods, or that there were changes in central VEGF that were not reflected in peripheral levels.

Behavioral research on progesterone has focused more on parental behavior than adult social behavior, but progesterone levels in marmosets have been associated with social grooming patterns, similar to the present findings (Azevedo et al., 2001). In addition, perinatal progesterone treatment in males has significant effects on adult social behavior (Hull et al., 1980). Immune studies of progesterone indicate that it inhibits both central and peripheral inflammation (Tait et al., 2008; Giannoni et al., 2011; Lei et al., 2014), and it is postulated that the decrease in CSS F2 male progesterone mediates the increase in GM-CSF and the decrease in social behavior. Based on earlier reports of the inhibitory actions of progesterone on maternal care (Bridges et al., 1978) and increased paternal behavior in male progesterone receptor KO mice and male mice treated with a progesterone antagonist (Schneider et al., 2003), future studies should investigate the effects of CSS on progesterone, the immune system, and maternal and paternal care.

The increased risk for psychiatric illness following exposure to early life stress may be mediated by enduring sex dependent changes in BDNF (Cirulli et al., 2009a,b). In mice, social deprivation, which may be similar to the depressed maternal care experienced by CSS F2 rats when young, decreases BDNF levels in the brain and increases anxiety (Berry et al., 2012). Exposure to low levels of maternal care in childhood is associated with increased DNA methylation of the BDNF and OXTR genes (Unternaehrer et al., 2015), and physical neglect and child abuse are associated with decreased plasma BDNF (Grassi-Oliveira et al., 2008; Elzinga et al., 2011). While studies of depression support the hypothesis that increases in inflammation result in decreased BDNF and attenuated neuroplasticity (Calabrese et al., 2014), we report decreases in both peripheral BDNF and inflammatory factors (although there is a trend for elevated IL-6 in CSS F2 females). In addition, although CSS decreased basal BDNF in F2 females, this change was not correlated with the decrease in allogrooming, suggesting that the difference in peripheral BDNF does not directly mediate the change in allogrooming. It may be that peripheral levels of BDNF do not reflect central levels that are more directly involved in mediating neuroplasticity and regulating behavior.

Alpha melanocyte stimulating hormone (α-MSH) mediates the immune response through the downregulation of pro-inflammatory cytokines, immunomodulatory cytokines, and costimulatory molecules (Lipton and Catania, 1997; Luger et al., 2003; Luger and Brzoska, 2007). This peptide, acting through MC4 receptors, is also involved in the etiology of depression and anxiety in animal models (Kokare et al., 2008, 2010; Liu et al., 2013). The stress of social isolation decreases social interaction and increases immobility in the forced swim test and treatment with HS014, a selective MC4 antagonist, attenuates depression symptoms (Kokare et al., 2010). Furthermore, HS014 has prophylactic actions on the adverse behavioral and neural effects of stress on depression and anxiety behaviors (Serova et al., 2013, 2014; Sabban et al., 2014). The elevated basal levels of α-MSH in the F2 males following transgenerational exposure to CSS may be adaptive in an inflammatory context by preventing excessive inflammatory responses, yet they may also increase susceptibility to stress induced depression and anxiety and disrupt social behavior.

In conclusion, the present study reports on several transgenerational treatment and sex dependent changes in the immune and hormonal profiles of the adult F2 offspring of dams exposed to CSS. Changes in α1AGP, GM-CSF, progesterone, and α-MSH are associated with transgenerational social stress induced decreases in allogrooming. These results support the hypothesis that transgenerational social stress affects both the immune system and social behavior, and also support previous studies on the adverse effects of early life stress on immune functioning and stress associated immunological disorders, including the increasing prevalence of asthma. The immune system may represent an important transgenerational etiological factor in disorders which involve social and/or early life stress associated changes in social behavior, such as depression, anxiety, and autism, as well as comorbid immune disorders. Future studies involving immune and/or endocrine assessments and manipulations will address specific questions of function and causation and may identify novel preventative measures and treatments for the growing number of immune mediated disorders.

## Figures and Tables

**TABLE 1 |:** Pearson Correlation coefficient and *p*-values for immune targets and hormones and allogrooming behavior during a social interaction test for factors significantly affected by chronic social stress in adult male and female F2 offspring of control (CON) and chronic social stress (CSS) dams.

Cytokine/Hormone	Group	Behavior	*r*	*r*^2^	*p*-value
**α1AGP**	**Male and female CON + CSS**	**Allogrooming**	**0.32**	**0.10**	**0.03**
**α1AGP**	**Female CON + CSS**	**Allogrooming**	**0.42**	**0.18**	**0.04**
α1AGP	Male CON + CSS	Allogrooming	−0.19	0.04	0.38
ICAM-1	Male and female CON + CSS	Allogrooming	0.11	0.01	0.43
ICAM-1	Female CON + CSS	Allogrooming	0.22	0.05	0.29
ICAM-1	Male CON + CSS	Allogrooming	0.09	0.01	0.69
GM CSF	Male and female CON + CSS	Allogrooming	0.02	0.003	0.91
GM CSF	Female CON + CSS	Allogrooming	−0.15	0.02	0.47
**GM CSF**	**Male CON + CSS**	**Allogrooming**	**−0.41**	**0.17**	**0.05**
BDNF	Male and female CON + CSS	Allogrooming	−0.09	0.008	0.54
BDNF	Female CON + CSS	Allogrooming	−0.001	0.000	0.99
BDNF	Male CON + CSS	Allogrooming	−0.08	0.006	0.72
α-MSH	Male and female CON + CSS	Allogrooming	−0.18	0.03	0.31
α-MSH	Female CON + CSS	Allogrooming	−0.11	0.01	0.52
**α-MSH**	**Male CON + ECSS**	**Allogrooming**	**−0.42**	**0.17**	**0.04**
**Progesterone**	**Male and female CON+CSS**	**Allogrooming**	**0.32**	**0.10**	**0.03**
Progesterone	Female CON + CSS	Allogrooming	0.05	0.003	0.79
Progesterone	Male CON + CSS	Allogrooming	−0.10	0.009	0.66

Bold values highlight significant associations.
